# Antimicrobial clerodane diterpenoids from *Microglossa angolensis* Oliv. et Hiern

**DOI:** 10.4103/0253-7613.51340

**Published:** 2009-04

**Authors:** J.D. Tamokou, J.R. Kuiate, M. Tene, P. Tane

**Affiliations:** 1 Laboratory of Microbiology and Antimicrobial Substances, Faculty of Science, University of Dschang, PO Box 67 Dschang, Cameroon; 2 Laboratory of Natural Product Chemistry, Faculty of Science, University of Dschang, PO Box 67 Dschang, Cameroon; 3 Department of Biochemistry, Faculty of Science, University of Yaoundé I, PO Box 812, Yaoundé, Cameroon

**Keywords:** Antimicrobial activity, chemical investigation, clerodane diterpenoids, compositae, *Microglossa angolensis*

## Abstract

**Objective:**

To identify the antimicrobial components present in *Microglossa angolensis* following fractionation of the methylene chloride extract of the aerial part of this plant.

**Materials and Methods:**

The plant was dried and extracted by percolation with methylene chloride. The dry extract was fractionated and purified by silica gel column chromatography. The isolated compounds were identified by comparison of their Nuclear Magnetic Resonance (NMR) spectral data with those reported in the literature. Antimicrobial activity was assayed by broth macro dilution method.

**Results:**

The crude extract of *M. angolensis* displayed significant antifungal and antibacterial activities (MIC = 312.50-1250μg/ml). 6β-(2-methylbut-2(Z)-enoyl)-3α,4α,15,16-bis-epoxy-8β,10βH-*ent*-cleroda-13(16),14-dien-20,12-olide and spinasterol were the most active compounds (MIC = 1.56-100μg/ml) and the most sensitive microorganisms were *Enterococcus faecalis* and *Candida tropicalis* for bacteria and yeasts respectively.

**Conclusion:**

The isolation of these active antibacterial and antifungal principles supports the use of *M. angolensis* in traditional medicine for the treatment of gastro-intestinal disorders.

## Introduction

*Microglossa angolensis* Oliv. et Hiern (Compositae) also known as *Conyza pyrrhopappa* Sch. Bip. ex A. Rich or *Microglossa pyrrhopappa* A. Rich., is an erect undershrub of 60 to 120 cm height distributed from south to tropical Africa and eastern Asia.[1,2] This plant is used traditionally to treat malaria and gastro-intestinal disorders in some African countries including Madagascar.[3] It is also used in the treatment of infections and wounds.[4] This suggests possible antimicrobial properties of this plant since some of the gastro-intestinal problems can be due to bacterial or fungal infections while wounds can be infected by bacteria. These facts prompted us to evaluate the methylene chloride extract of this plant and two of its clerodane diterpenoids for their antibacterial and antifungal activities.

In fact, over the last three decades there has been an increase in opportunistic fungal infections, including life-threatening invasive mycoses as well as bacterial infections due to the high prevalence of immune-suppressing disease conditions such as HIV-AIDS, organ transplantation, cancer and multidrug resistance problems observed mainly with bacteria.[5] In developing countries, this situation is more serious, due to many factors like the economic crisis, high cost of industrialized medicines and inefficient healthcare systems.

Indeed, the search for new antifungal and antibacterial agents from natural sources has intensified in response to the limitations of currently available therapy and the emergence of drug-resistant strains. It is important to evaluate these extracts for a possible standardization to overcome the empirical use. The present study investigates the *in vitro* antibacterial and antifungal properties of methylene chloride extract of *M. angolensis* and three of its isolated pure compounds against some bacteria and yeasts.

## Materials and Methods

### General experimental procedures for structure elucidation

Melting points (uncorr.) were determined on a Kofler apparatus. Optical rotations were measured on a AA Series Automatic Polarimeter Polaar-2000 at 22°C. ^1^H NMR (400.13 MHz) and ^13^C NMR (100.6 MHz) with DEPT program were recorded at room temperature in CdCl_3_, unless otherwise stated, using a Bruker DPX 400 spectrometer. COSY, HMQC and HMBC experiments were recorded with gradient enhancements using sine shape gradient pulses. The IR spectra were recorded with a Shimadzu FT-IR-8400S spectrophotometer and the UV spectra recorded with a Shimadzu UV-3101 PC spectrophotometer. CIMS, HRCIMS spectra were recorded with a JEOL JMS-700 spectrometer. Column chromatography [CC] was run on Merck silica gel 60 and gel permeation on sephadex LH-20, while thin layer chromatography (TLC) was carried out either on silica gel GF_254_ pre-coated plates (analytical TLC) or on silica gel 60 PF_254_ containing gypsum (preparative TLC), with detection accomplished by spraying with 50% H_2_SO_4_ followed by heating at 100°C, or by visualizing with an UV lamp at 254 and 366 nm.

### Plant material

The aerial parts of *M. angolensis* were collected in Dschang, West Province, Cameroon, in September 2006. Authentication was confirmed by Mr. François Nana, a botanist of the Cameroon National Herbarium, Yaoundé. A voucher specimen (BUD 0630) was deposited at the Department of Botany, University of Dschang.

### Preparation of plant extract and fractionation

The air-dried powdered material (2.5 kg) was extracted by percolation with CH_2_ Cl_2_ for three days at room temperature. Removal of the solvent under reduced pressure provided 70 gm (2.80% w/w) of a greenish organic extract. A portion (60 gm) was subjected to CC on silica gel (70-230 mesh) and gradient elution performed with mixtures of hexane and ethyl acetate. Fifty-five fractions of 250 ml each were collected and combined on the basis of their TLC profiles to give 6 major fractions: I (18 gm, hexane-EtOAc 100:0 and 19:1), II (8 gm, hexane-EtOAc 19:1), III (4 gm, hexane-EtOAc 9:1 and 4:1), IV (2.6 gm, hexane-EtOAc 7:3), V (10 gm, hexane-EtOAc 7:3 and 1:1) and VI (8.7 gm, EtOAc). Further purification of these fractions was achieved separately by silica gel column chromatography by gradient elution with hexane-EtOAc. Fraction I yielded only straight chain fatty alcohols, while fraction II gave β-amyrin (70 mg) and other fatty alcohols. Fraction III afforded spinasterol (120 mg). Fraction IV yielded 6β-(2-methylbut-2(Z)-enoyl)-3α,4α,15,16-bis-epoxy-8β,10βH-*ent* -cleroda-13(16),14-dien-20,12-olide (14 mg). Fraction V afforded 10β-hydroxy-6-oxo - 3α,4α,15,16-bis-epoxy-8βH-cleroda-13(16),14-dien-20,12-olide (106 mg) and a mixture of two flavones (42 mg) which were separated by preparative TLC (eluent: CH_2_Cl_2_-MeOH, 98:2) to give 5,7-dihydroxy-3,8,3',4'-tetramethoxyflavone (10 mg) and 5,7-dihydroxy-3,8,3',4',5'-pentamethoxyflavone (6 mg). The last fraction (VI) yielded straight chain fatty acids and a complex mixture. For the individual fractions, an additional purification by CC on LH-20 gel eluted with CH_2_Cl_2_-MeOH (1:1) was required to obtain analytically pure samples (this removed the last traces of chlorophyll).

### Microorganisms

The microorganisms used in this study consisted of three bacteria (*Enterococcus faecalis* ATCC 27853, *Staphylococcus aureus* ATCC 25922 and *Salmonella typhi* ATCC 6539) and three fungi (*Candida albicans* ATCC 9002, *Candida parapsilosis* ATCC 22019 and *Candida tropicalis* ATCC 750) reference strains obtained from the American Type Culture Collection (ATCC). Also, one clinical isolate of bacteria (*Proteus mirabilis*) collected from the Pasteur Centre (Yaoundé-Cameroon) was used. All strains of bacteria and yeasts were grown at 35°C and maintained on nutrient agar (NA, Conda) and Sabouraud Dextrose Agar (SDA, Conda) slants respectively.

### Antimicrobial assay

The minimum inhibitory concentrations (MICs) were determined by broth macro dilution method with slight modifications from the one described by Gulluce *et al*.[6] The two-fold serial dilutions in concentration of the extract (2.50-0.078 mg/ml) and pure products (400-0.19*μ*g/ml) were prepared in Mueller Hinton Broth (MHB, Conda) for bacteria and Sabouraud Dextrose Broth (SDB, Conda) for yeasts. The inocula of microorganisms were prepared from 24 h old broth cultures. The absorbance was read at 600 nm and adjusted with sterile physiological solution to match that of a 0.5 McFarland standard solution. From the prepared microbial solutions, other dilutions with sterile physiological solution were prepared to give a final concentration of 10^6^ colony-forming units (CFU) per milliliter for bacteria and 5 × 10^5^ spores per milliliter for yeasts. For every experiment, a sterility check (5% v/v aqueous DMSO and medium), negative control (5% v/v aqueous DMSO, medium and inoculum) and positive control (5% v/v aqueous DMSO, medium, inoculum and water-soluble antibacterial or antifungal antibiotic) were included. In general, the 24-macro well plates (Nunclon) were prepared by dispensing into each well 880*μ*l of an appropriate medium, 100*μ*l of test substances (crude extract or pure products) and 20*μ*l of the inoculum (10^6^ CFU/ml for bacteria and 5 × 10^5^ spores/ml for yeasts). The content of each well was mixed thoroughly with a multi-channel pipette and the macro well plates were covered with the sterile sealer and incubated at 35°C for 24 h (for bacteria) and 48 h (for yeasts) under shaking by using a plate shaker (Flow Laboratory) at 300 rpm. Microbial growth in each well was determined by observing and comparing the test wells with the positive and negative controls. The absence of microbial growth was interpreted as antibacterial or antifungal activity. The MIC was the lowest concentration of the test substances that prevented visible growth of microorganisms. Minimum Bactericidal Concentrations (MBCs) or Minimum Fungicidal Concentrations (MFCs) were determined by plating 10*μ*l from each negative well and from the positive growth control on Mueller Hinton Agar (for bacteria) and Sabouraud Dextrose Agar (for yeasts). MBCs or MFCs were defined as the lowest concentration yielding negative subcultures or only one colony. All the experiments were performed in triplicate. Gentamicin (Sigma) and Nystatin (Sigma) at the concentration ranging between 400 and 0.78*μ*g/ml served as positive controls for antibacterial and antifungal activities respectively.

## Results

**Figure 1:**
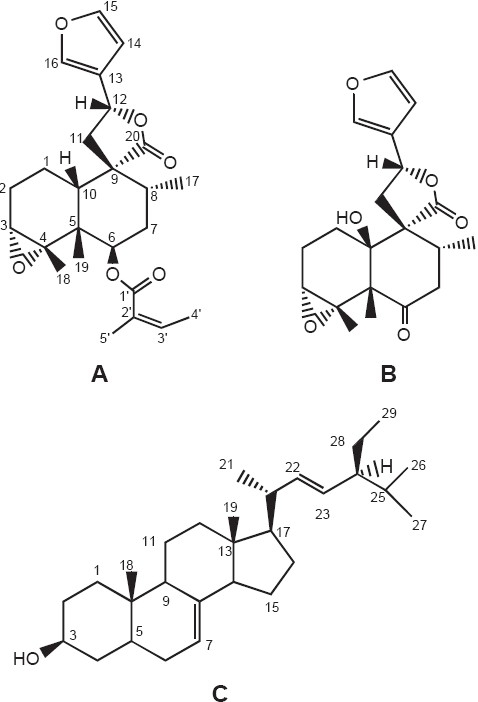
Chemical structures of 6β-(2-methylbut-2(Z)-enoyl)-3α,4α,15,16-bis-epoxy-8β,10βH-*ent*-cleroda-13(16),14-dien-20, 12-olide (A), 10β-hydroxy-6-oxo - 3α,4α,15,16-bis-epoxy-8βH-cleroda- 13(16),14-dien-20,12-olide (B) and spinasterol (C)

Six compounds were isolated from the methylene chloride extract of aerial part of *M. angolensis*. Their structures were elucidated on the basis of spectroscopic data (IR, CIMS, ^1^H-NMR and ^13^C-NMR, HMQC, HMBC). These compounds are 6β-(2-methylbut-2(Z)-enoyl)-3α,4α,15,16-bis-epoxy-8β, 10βH-*ent*-cleroda-13(16),14-dien-20,12-olide[7] (A) and 10β-hydroxy-6-oxo - 3α,4α,15,16-bis-epoxy-8βH-cleroda-13(16),14-dien-20,12-olide[7] (B), spinasterol[8] (C), β-amyrin,[9] 5,7-dihydroxy-3,8,3',4'-tetramethoxyflavone[10] and 5,7-dihydroxy-3,8,3',4',5'-pentamethoxyflavone.[11] The crude extract and three of the isolated compounds A, B and C [Figure 1] were tested for their antibacterial and antifungal properties [Table 1]. 5,7-dihydroxy-3,8,3',4'-tetramethoxyflavone and 5,7-dihydroxy-3,8,3',4',5'-pentamethoxyflavone were not tested due to their minor quantity. The crude extract as well as compounds A and C showed both antifungal and antibacterial activities. Compounds A and C were more effective on gram (+) compared to gram (−) bacteria. Their MICs were even lower than that of reference compound Gentamicin. Compound B did not present any antibacterial activity but very low antifungal activity on *C. parapsilosis* and *C. tropicalis*.

**Table 1:** Inhibition parameters (MIC, MBC and MFC) of methylene chloride extract of *M. angolensis* and some of its isolated constituents (μg/ml)

*Microorganisms*	*Parameters*	*Test substances*					
		
		*CH_2_Cl_2_ extract*	*A*	*B*	*C*	*Gentamicin*	*Nystatin*
Bacteria							
*Salmonella typhi*	MIC	312.50	na	na	na	50	/
	MBC	625	/	/	/	50	
	MBC/MIC	2	/	/	/	1	
*Staphylococcus aureus*	MIC	625	6.25	na	6.25	50	/
	MBC	625	12.50	/	12.50	50	
	MBC/MIC	1	2	/	2	1	
*Enterococcus faecalis*	MIC	1250	3.12	na	1.56	12.50	/
	MBC	1250	6.25	/	1.56	12.50	
	MBC/MIC	1	2	/	1	1	
*Proteus mirabilis*	MIC	625	25	na	50	100	/
	MBC	1250	50	/	50	100	
	MBC/MIC	2	2	/	1	1	
Yeasts							
*Candida albicans*	MIC	1250	100	na	50	/	1.56
	MFC	1250	100	/	50		1.56
	MFC/MIC	1	1	/	1		1
*Candida parapsilosis*	MIC	625	50	400	25	/	12.50
	MFC	625	100	400	50		12.50
	MFC/MIC	1	2	1	2		1
*Candida tropicalis*	MIC	312.50	12.50	200	6.25	/	6.25
	MFC	625	50	200	12.50		6.25
	MFC/MIC	2	4	1	2		1

/: Not tested; A: 6β-(2-methylbut-2(Z)-enoyl)-3α,4α,15,16-bis-epoxy-8β,10βH-*ent*-cleroda-13(16),14-dien-20,12-olide; B: 10β-hydroxy-6-oxo - 3α,4α,15,16-bis-epoxy-8βH-cleroda-13(16),14-dien-20,12-olide; C: Spinasterol; na: Not active at concentrations up to 400 μg/ml

## Discussion

All the isolated compounds were previously described in the literature.[7–11] For the antimicrobial properties, we focused our attention on the crude extract, two clerodane diterpenoids (compounds A and B) and spinasterol (Compound C). Clerodane diterpenoids are known to possess anti-tumoral, antibacterial and antifungal properties.[12–14] Compound A was more effective on Gram (+) bacteria than Gram (−) and with MICs lower than that of reference antibacterial Gentamicin. This observation is in agreement with data mentioned by some other authors as far as clerodane diterpenoids are concerned.[13–17] In contrast, compound B, with the same basic skeleton, showed no antibacterial activity. This can be attributed to additional 2-methyl but-2 (Z)-enoyl group at position 6 and the absence of hydroxyl group at position 10 in compound A. It is also interesting to note that compound A does not contain acidic group like other clerodane diterpenoids having the same range of antibacterial activities. According to Urzúa *et al.*,[14] two major structural requirements are to be fulfilled for the antibacterial activity of these compounds: The presence of a hydrophobic skeleton and a lipophilic side chain with a free acid group. In fact, in compound A, the acidic group is esterified and the additional 2-methyl but-2 (Z)-enoyl substitute can explain the biological activity of this compound. Murthy *et al*.[13] also mentioned clerodane diterpenoids that possess antibacterial activity but no free acidic group. Compound C possesses relatively good antimicrobial activity against yeast and bacteria. This is not surprising since individual triterpenes have shown this type of biological activities.[18] According to Cowan,[19] terpenes may have the ability to disrupt microbial membrane and this may explain their antimicrobial properties. Our data showed that the response in terms of susceptibility to tested drugs varied among the strains. The differences in susceptibility may be explained by differences in cell wall composition and/or genetic content of plasmids that can be easily transferred among microbial strains.[20] For all the tested compounds, the ratios MBC/MIC were less than or equal to 2 and thus they can be considered bactericidal. According to Carbonnelle *et al.*,[21] a compound with MBC/MIC ≤ 4 is to be considered bactericidal while a compound with MBC/MIC > 4 is bacteriostatic. Finally, none of the compounds A, B and C showed anti-salmonella activity while the crude extract was active against *Salmonella typhi*. It is possible that the active principle on this microorganism was different from the one tested or this activity was a result of synergetic effects between some constituents of the crude extract.

In conclusion, we consider that *M. angolensis* is a promising antibacterial and antifungal species. In addition, we have found that 6β-(2-methylbut-2(Z)-enoyl)-3α,4α,15,16-bis-epoxy-8β,10βH-*ent*-cleroda-13(16),14-dien-20,12-olide and spinasterol are the most active compounds while the most sensitive microorganisms were *Enterococcus faecalis* and *Candida tropicalis* for bacteria and yeast respectively. The results justified the traditional use of this plant to cure infectious diseases. However, further study is required to evaluate the effect and toxicity of these compounds in experimental animals and to establish if they could be safely used as a topical antimicrobial agent.
